# Tortuosity of Aligned Channels in Alumina Membranes Produced by Vacuum-Induced Surface Directional Freezing

**DOI:** 10.3390/ma10040409

**Published:** 2017-04-14

**Authors:** Sandra Großberger, Tobias Fey, Geoffrey Lee

**Affiliations:** 1 Division of Pharmaceutics, University of Erlangen, 91054 Erlangen, Germany; sandra.grossberger@fau.de; 2 Department of Material Science and Engineering, University of Erlangen, 91054 Erlangen, Germany; Tobias.Fey@fau.de

**Keywords:** alumina, directional freezing, tortuosity

## Abstract

Vacuum-induced surface freezing of colloidal alumina was used to produce membranes that have elongated, aligned channels and, hence, are tortuous in the direction perpendicular to ice crystal growth. The effective tortuosity of the membranes was measured by steady-state diffusion of a solute, methylene blue. The resulting diffusion profiles show an initial step-increase in amount of dye reaching the acceptor that is caused by capillarity drawing the donor solution through any non-wetted channels in the membrane. This is followed by a linear steady-state phase whose flux is proportional to dye concentration in the donor and inversely proportional to the colloid’s volume fraction of dispersed phase. From the steady-state flux, the effective tortuosity, τ* = (*α*/*τ*)^−1^, was calculated. This is the reciprocal quotient of the reduced available area for diffusion within the membrane, α = A*/*A*, where A* is the available area and *A* is the cross-sectional area of the membrane, and the increased mean diffusional path length, i.e., tortuosity $=L^{*}/L$, where L* is the mean path length and *L* is the membrane thickness. The values of τ* lie in the range of 2–38 and increase as the volume fraction of dispersed phase is larger. This latter effect indicates that τ* > 1 results, to a larger extent, from the reduced available diffusion area, *α*, than from the lengthened pathway, *τ*, in these aligned porous membranes.

## 1. Introduction

In our previous work [1] we developed the specialized technique of vacuum-induced surface freezing [2] to produce aligned porous alumina monoliths that have parallel channels running through the solid from top to bottom. This is an alternate technique to the freeze-casting methods used before which require a specialized cold-finger apparatus [3,4,5,6,7,8,9]. A vacuum of approximately 250 mTorr produces undercooling of the dispersion surface, which freezes to form a continuous, frozen surface layer. Subsequent sublimation keeps this surface cold and directional freezing occurs from top to bottom through the dispersion [9]. Since this is done in a glass vial standing on the shelf of a lyophilizer, it does not need a complex cold-finger freezing apparatus. The aligned porous morphology of the lyophilized ceramic is highly similar to the structures produced by conventional freeze casting. A more-or-less dense surface layer and cellular sub-surface region border a wide region of aligned channels that stretches through most of the depth of the membrane [1].

Aligned porous materials can be used as scaffolds for tissue engineering, e.g., synthetic bone generation on hydroxyapatite, because of their high compressive strengths [3,10]. There is, however, a further potential application as porous membranes for use in diffusion and separation processes. One particular application is the provision of highly-tortuous membranes for use as a surrogate for human stratum corneum. This is the outermost layer of the skin and of relevance for testing transdermal patches that deliver drugs through the skin into the systemic circulation [11]. No synthetic membrane has a tortuosity sufficiently high to simulate that of the stratum corneum [12]. This application is governed by the particular orientation of the linearly-aligned channels to the direction of solute or solvent movement through the membrane. In the direction parallel to the channels the membrane’s tortuosity, τ, will be very close to unity [13], i.e., $\tau =L^{*}/L$ ≈ 1.0, where $L^{*}$ is the mean path (pore) length and L is the membrane thickness measured parallel to the aligned pores [14]. This is because the aligned channels run linearly straight through the thickness of the membrane from top to bottom. In the direction perpendicular to the channels, however, the tortuosity should be larger than 1.0. The reason is that to pass sideways through the membrane, e.g., from left to right, the movement of solute or solvent must occur on a convoluted pathway that circumvents the solid lamellae walls separating the channels. This requires the existence of cross-linking pores through the lamellae that connect adjacent parallel channels which have, however, been identified in alumina monoliths made by vacuum-induced surface directional freezing [1].

We describe, in this paper, our efforts to measure the tortuosity of alumina membranes produced by vacuum-induced surface directional freezing. The wide region of aligned channels formed by freezing, lyophilization, and sintering was isolated and used as a membrane. The steady-state diffusion of a model solute, methylene blue, in the direction perpendicular to ice crystal growth was measured to identify if a continuous channel pathway exists. From the measured diffusion rate it is readily possible to calculate the effective tortuosity of the membrane [15], (*α*/*τ*)^−1^, from:

(1) $$\frac{\Delta m\left(t\right)}{A\Delta t}=\frac{Dc_{0}}{L}\cdot \frac{\alpha }{\tau }$$

where (*α*/*τ*)^−1^ is seen to be the factor by which the solute flux, Δ*m*(*t*)/Δ*t*, out of a donor solution of concentration *c*_0_ through the tortuous membrane, is reduced compared with that through an isotropic membrane of the same area, *A*, and thickness, *L*, with solute diffusivity, *D*. (*α*/*τ*)^−1^ is, therefore, the reciprocal value of the quotient of the reduced available area for diffusion within the membrane, α = A*/*A*, where A* is the available area and *A* is the cross-sectional area of the membrane, to the increased mean diffusional path length, i.e., tortuosity, $\tau =L^{*}/L$. It was, therefore, the object of this study to determine the effective tortuosity of these aligned porous membranes.

## 2. Results and Discussion

**Figure 1:**
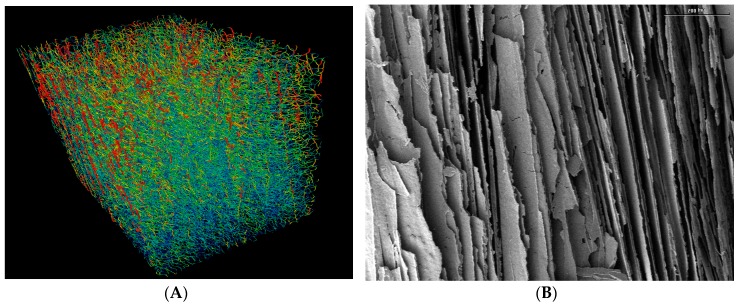
Images of the sintered membrane prepared from alumina dispersion at volume fraction of dispersed phase, φ_v_ = 0.128. (**A**) Pore network/channel distribution of section determined with micro-computed tomography. The pore network is represented in red to indicate maximum pore diameter and blue for minimum pore diameter; (**B**) Scanning electron micrograph of the longitudinal section.

Figure 1A shows the pore channels and their distributions in the sintered membrane obtained from the example of the alumina dispersion having a volume fraction of dispersed phase of φ_v_ = 0.128. The pore network is represented with a red color to indicate maximum pore diameter and a blue color for minimum pore diameter. For ease of visualization the true channel diameter is reduced by a factor of 0.6 to enable an improved insight view. The channels are predominantly aligned in parallel and run from top to bottom of the sample. As far as can be judged by eye, the channels appear to be continuous between the top and bottom of the membrane. The tortuosity of this membrane perpendicular to the direction of freezing (top to bottom) should be >1.0. The SEM picture in Figure 1B is a longitudinal section through the region of aligned channels within the same membrane (φ_v_ = 0.128). Additionally, in this image the parallel arrangement of aligned solid walls and channels runs through the depth of the membrane and is the same geometry at all three φ_v_ [7]. The diffusion of methylene blue took place perpendicular to the direction of the aligned channels, i.e., in this image from left to right. Note the presence of pores and cracks in the walls in Figure 1B. These become increasingly evident as φ_v_ is reduced from 0.128 (compare Figure 1B) through 0.064 to 0.032 (SEMs not shown) [1].

**Figure 2:**
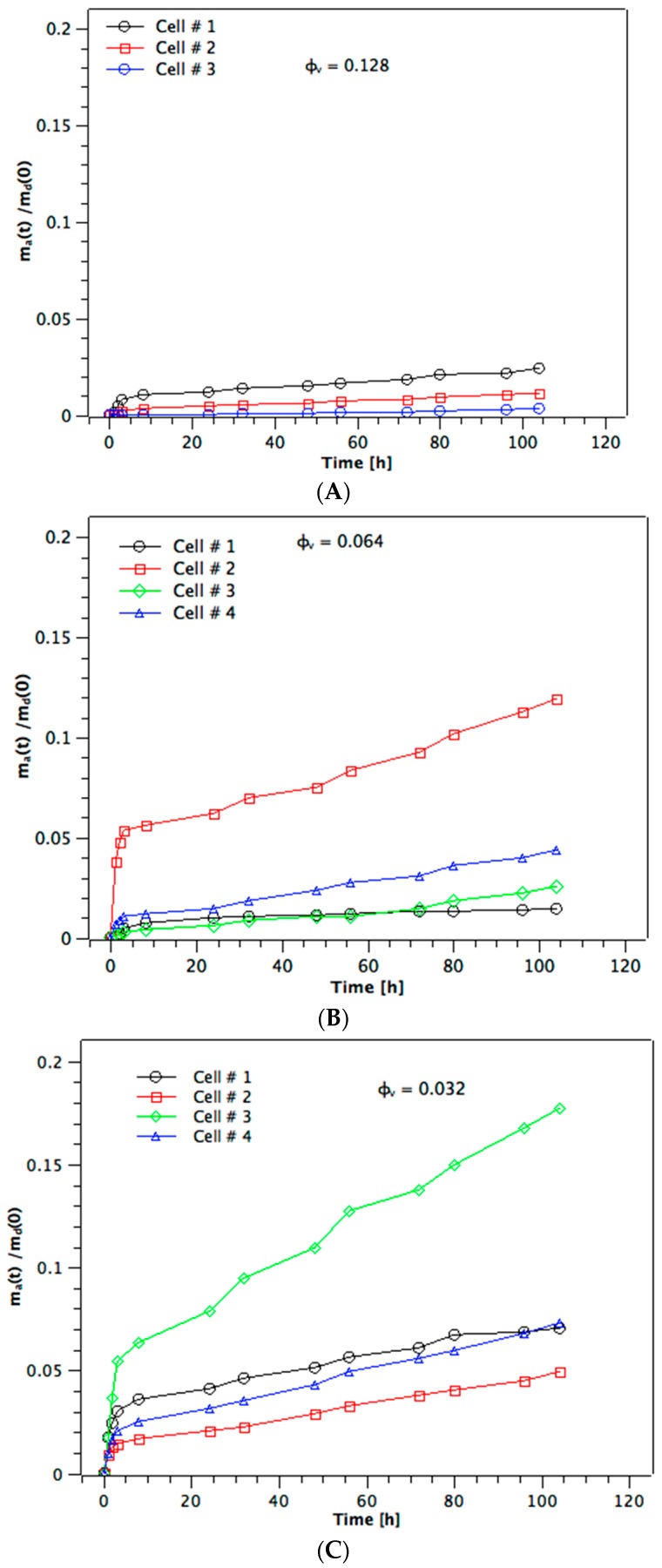
Diffusion profiles of m_a_(*t*)/m(0) versus t for individual diffusion experiments using aligned porous membranes prepared from alumina slurries at different volume fractions of the dispersed phase, φ_v_. (**A**) φ_v_ = 0.032; (**B**) φ_v_ = 0.064; and (**C**) φ_v_ = 0.128. m_a_(*t*) is the amount of dye detected in the acceptor phase at time *t*, and m_d_(0) is the initial amount present in the donor phase.

Figure 2A–C show the diffusion profiles for methylene blue obtained from a 0.20 mg/5 mL donor solution, i.e., m_d_(0) = 0.2 mg methylene blue, for membranes made from the three different φ_v_. We give the individual profiles (*n* = 3 for Figure 2A—one cell failed—and *n* = 4 for Figure 2B,C) rather than mean ± standard deviation since this helps understand better the variation seen at earlier times. The diffusion profiles at both the higher and lower m_d_(0), i.e., of 0.1 mg and 0.3 mg, respectively, show the same pattern (not given here for brevity). It is immediately evident that the diffusion profiles do not show the expected shape for membrane permeation with a lag time followed by a linear steady-state phase [16]. The amount of dye appearing in the acceptor solution shows a rapid initial step-increase during the first 5 h, after which it levels off and shows an approximately linear further increase up to the end of the experiment at 104 h. This behavior is the same at all φ_v_, the only difference being that the magnitudes of both the initial step-increase in m_a_(*t*) and the subsequent linear slopes are larger at lower values of φ_v_. Furthermore, although the magnitude of the initial step varies substantially within the 3–4 membranes tested at any single φ_v_, the linear slope is more consistent at given volume fraction of dispersed phase. At the lowest φ_v_ of 0.032 (Figure 2C) the value of m_a_(*t*)/m_d_(0) reaches, at most, 0.18 = 18% of the total mass of dye in the initial donor solution, with three out of four profiles only reaching 0.07. At both the higher φ_v_ the maximum values reached for m_a_(*t*)/m_d_(0) are 0.12 and 0.025. The near–linear second phase of the diffusion profiles is, therefore, a steady-state [17].

The rapid initial step-increase cannot come from a diffusion-controlled transport process through the sintered membranes. Even if the channels were oriented horizontally straight through the membrane from the donor to the acceptor, i.e., *τ* = 1, the diffusion lag time, *t_lag_* = $L^{*}$^2^/6D_meth_, is calculated to be 1.8 h. Indeed, detailed micro-computer tomography has shown that the channels in these aligned porous membranes run in domains that are perpendicular to the direction of diffusion [1], and the tortuosity is, therefore, expected to be larger than 1.0. This is also the impression given by the image in Figure 1A. If we assume *τ* = 5, for example, the calculated value of *t_lag_* rises to 46 h. It is, therefore, highly unlikely that the initial step-increase in m_a_(*t*) comes only from diffusion through the channels. Up to 1% of the total mass of dye is already detected in the acceptor at the first sampling point of *t* = 1 h.

It is plausible that the initial step-increase is a result of capillarity through the aligned porous membrane. The Laplace capillary pressure will draw the donor solution through non-wetted channels and into the acceptor as soon as the donor chamber of the diffusion cells is filled with dye solution. This behavior can be approximated by the Washburn equation [17]. The effects of atmospheric and hydrostatic pressure can be neglected. In this case the rate of liquid movement through a cylindrical channel of radius, *r*, is [18]:

(2) $$\frac{dl\left(t\right)}{dt}=\frac{\gamma_{lv}\cdot rcos\theta_{ls}}{4\cdot \eta \cdot l}$$

where *l*(*t*) is the distance penetrated into the channel after time, *t*, $\gamma_{lv}$ is the liquid/air interfacial tension, *θ_ls_* is the liquid/solid contact angle and *η* is the liquid dynamic viscosity. Integration of Equation (2) and solving for *t* gives:

(3) $$\hat{t}=\frac{2\cdot \eta \cdot \hat{l}{\left(t\right)}^{2}}{\gamma_{lv}\cdot rcos\theta_{ls}}$$

where $\hat{t}$ is the time it takes for the liquid’s meniscus to travel through the channel of length $\hat{l}$. The channel radius is taken as 40 µm which was determined from micro computer tomography of cross sections through the aligned region of the membrane [1]. Note that the validity of Washburn in such fine channels (down to 3 µm radius) has been demonstrated with water [19]. The contact angle of water on sintered alumina compacts, *θ_ls_*, is 37° [20]. For an assumed tortuosity of *τ* = 1 the calculated value for $\hat{t}$ using Equation (2) is 50 ms. For an assumed *τ* = 5, the $\hat{t}$ lengthens to 0.86 s and for assumed *τ* = 10 to 4.5 s. It is, therefore, clear that capillarity through the very fine aligned channels in the membrane is rapid. This was confirmed by observing the almost instantaneous uptake of a droplet into the surface of a membrane (result not shown). We suggest that this is the cause of the step-increase in m_a_(*t*) observed in the first time-samples. The capillary pressure falls to zero once the donor liquid passing through the channel reaches the acceptor solution and further dye transport can then only occur by diffusion. The reason for the non-wetting of some channels is unclear but could be caused by the complex interconnected geometry of the channel system as observed by SEM and µ*C*_t_ [1].

**Figure 3:**
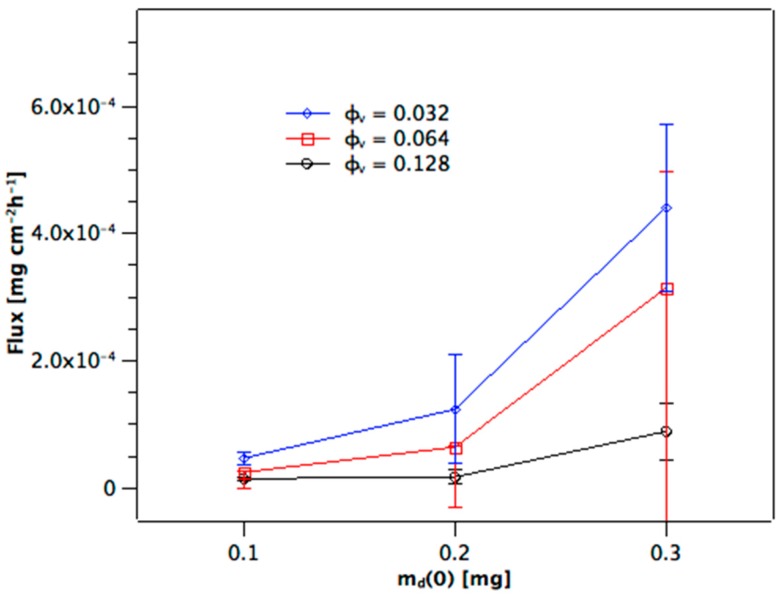
Dependence of stead-state flux, Δm_a_(*t*)/Δ*t*, on initial dye loading of donor, m_d_(0), and volume fraction of dispersed phase, φ_v_.

**Figure 4:**
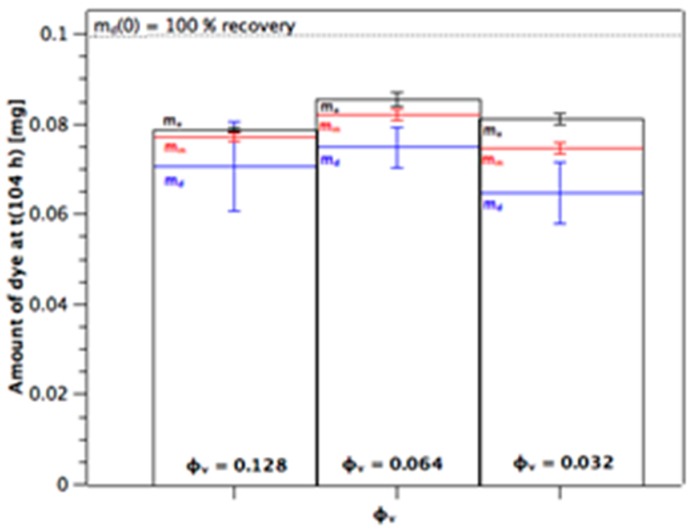
Measured distribution of dye at end of experiment between donor, m_d_(104 h), membrane, m_m_(104 h) and acceptor, m_a_(104 h), for the m_d_(0) = 0.1 mg series. Values are mean averages ± standard deviation (*n* = 3 or 4).

Once the near-linear steady-state phase has been reached at *t* ≈ 10 h, the slopes are fairly uniform for the membranes from a given φ_v_ (cf. Figure 2A–C). The values of the slope give the flux of the steady-state diffusion through the channels of the membrane, Δm_a_(*t*)/*A*Δ*t*. Figure 3 shows that flux increases over-proportionally at higher m_d_(0), whereas this is expected to be linear from Equation (1). Additionally, the flux increases as the volume fraction of dispersed phase of the alumina colloid used to prepare the membrane is reduced. This is because by decreasing φ_v_ the porosity of the sintered membrane becomes larger. This must increase the effective area available for diffusion within the membrane. How it likely changes the path length, which will be discussed below. Figure 4 shows the distribution of the dye between donor (blue), membrane (red), and acceptor (black) as measured at the end of the experiment for the m_d_(0) = 0.1 mg series. The amount detected at the acceptor, m_a_(104 h), is larger as the volume fraction decreases because of the higher flux (cf. Figure 3). There is also a slight trend to higher amounts of dye in the membrane as the volume fraction decreases which would be the case for higher porosity. Note that the total recoveries lie below 100%. We suggest that the reason for this is the very high relative donor concentration; a small analytical error in m_d_(104 h) represents a high amount of dye compared to the amounts present in membrane and acceptor. The recoveries with both the m_d_(0) = 0.2 mg and 0.3 mg series were 90%–105% (not shown), with the distributions being very similar to that shown in Figure 4 for the m_d_(0) = 0.1 mg series.

**Figure 5:**
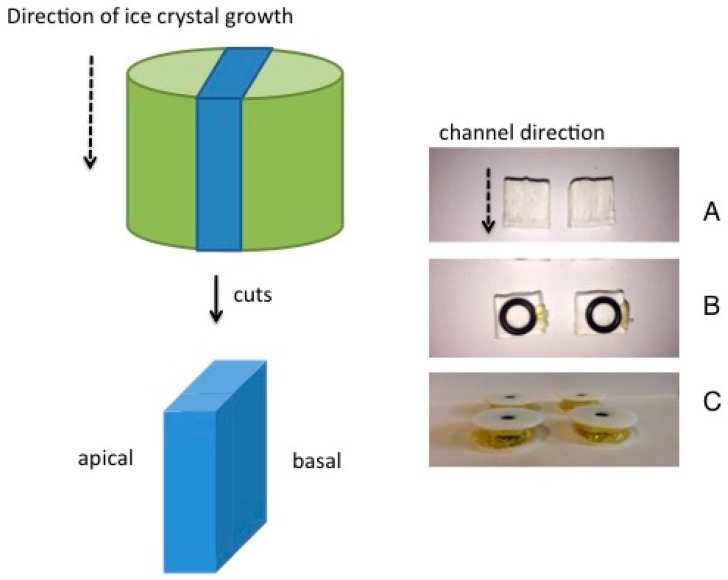
Preparation of membrane (colored blue) from sintered body (colored green). (**A**) Laminar membranes after cutting from sintered body; (**B**) After positioning a rubber sealing ring onto apical side of membrane; (**C**) Complete membrane contained between two circular washers.

Note that the position of the membrane held between the two halves of the diffusion cell will result in radial diffusion of the dye out to the edge regions of the membrane that lie outside of the rubber sealant ring and have no direct contact to donor or receiver solutions (cf. Figure 5B). Although this effect will increase the diffusional lag-time, *t_lag_*, especially for a thick membrane, such as that used here (*L* = 0.5 cm), its effects on steady-state flux will be marginal [21]. Equation (1) is now used to calculate the effective tortuosity τ* = (*α*/*τ*)^−1^ from the measured values of Δm_a_(*t*)/Δ*t*. Recall that τ* is the factor by which the solute flux through a tortuous membrane is reduced compared with that through an isotropic membrane [15], i.e., $\alpha /\tau =\left(A^{*}\cdot L\right)/\left(A\cdot L^{*}\right).$ The value for D_meth_ of methylene blue in water was taken from the literature. Table 1 gives the calculated values for τ* that were each measured at three donor loadings, m_d_(0), and at the different φ_v_.

The results for τ* illustrate a couple of points. First, the values for τ* are substantially higher than unity. It follows that either the tortuosity in the membrane, *τ*, is >1.0, or the surface area factor, *α*, <1.0, or a combination of both factors exists. Scanning electron microscopy has shown a parallel alignment of channels and solid walls through the membrane from top to bottom [1]. When cut and placed within the diffusion cell, the direction of solute diffusion is perpendicular to the run of the channels. Since the sintered alumina walls may be assumed to be impermeable, it follows that the diffusional path-length through the membrane must be longer than the membrane’s outer thickness. The presence of these walls between the channels must also reduce the area available for diffusion within the membrane. The structural information available from SEM and µCT suggests, therefore, that the values for τ* are a combination of both path length and area factors.

Secondly, for the membranes prepared from a higher volume fraction of dispersed phase, φ_v_, the values for τ* are greater. The µCT data for these membranes showed that higher φ_v_ produced thicker walls between the channels [1]. This effect as also been observed for alumina slurries that were freeze cast between two cold-fingers [22] rather than vacuum-induced surface frozen, as in the current work. If the walls between the channels become thicker, volume constraints mean that the diffusion path length through the channels cannot increase. It follows that $\tau =L^{*}/L$ cannot be larger at higher φ_v_; if anything, it should become smaller. It seems plausible that higher φ_v_ decreases primarily the value of α = A*/*A*, with the result that τ* becomes larger. Indeed, the SEM data showed that higher φ_v_ produced denser walls between the channels, which have fewer perforations and cracks than at lower alumina concentrations [7].

## 3. Materials and Methods

### 3.1. Materials

Aluminum oxide powder, Al_2_O_3_ CT 3000 SG (Almatis, Ludwigshafen, Germany) has a specific surface area of 7.8 m^2^/g and a mean particle diameter of 500 nm. Polyvinyl alcohol of molecular weight 21,000 (PVA; Mowiol 4-88; Sigma-Aldrich, Munich, Germany), ammonium polymethacylate anionic dispersant (Darvan CN, RT Vanderbilt Co., Norwalk, CT, USA), and Tego Airex 901W defoaming agent (Evonik, Frankfurt, Germany) were used as received. Methylene blue was obtained from Sigma-Aldrich (Munich, Germany). The adhesive used to seal the side walls of the cut membrane was Hot Adhesive Pattex Hot Sticks (Heinkel AG, Düsseldorf, Germany). Water was double-distilled from an all-glass apparatus. 

### 3.2. Preparation of Sintered Alumina Membranes

This has been described fully before [1]. Briefly, dispersions were prepared by dispersing the Al_2_O_3_ powder in water to volume fractions, φ_v_, of 0.128, 0.064 and 0.032 (equivalent to concentrations of 50%, 25% and 12.5% *w*/*w*, respectively). The total solids’ content also contained 0.7% *w*/*w* PVA/DARVAN as a binding/dispersing agent and 0.175% *w*/*w* TEGO Airex to prevent excessive foaming under vacuum. Each dispersion was stirred for 24 h at room temperature before being transferred as 3 mL portions into 5 mL glass vials (Carl Roth, Karlsruhe, Germany). The vials were then placed on a precooled shelf at shelf temperature, T_shelf_, of +10 °C in a Virtis Genesis 25 EL lyophilizer. After 35 min the chamber pressure, P_cham_, was reduced to 150 mTorr (≈0.2 mbar) to induce freezing of the dispersion surface and subsequent ice crystal growth down through the dispersion from top to bottom [8]. On completion of directional freezing T_shelf_ was reduced at 10 °C·min^−1^ to −18 °C to prevent ice melting and P_cham_ reduced to 50 mTorr (=0.07 mbar) during primary drying. When the difference between Pirani and capacitance manometers reached ≤3 mTorr the secondary drying phase was started by increasing T_shelf_ at 0.12 °C/min over 6 h and holding P_cham_ at 50 mTorr. At the end of the lyophilization cycle the chamber was flooded with dry nitrogen gas. Each green body was removed from its glass vial and sintered in an air furnace (Hochtemperaturofen HT16/16, Nabertherm GmbH, Lilienthal, Germany) for 2 h. The furnace temperature started at 400 °C to burn off the PVA and produce debinding of the colloidal alumina particles. Further heating to the sintering temperature of 1700 °C was then done at 300 °C/h. After the 2 h sintering at 1700 °C was complete, the cooling rate was 300 °C/h down to room temperature. 

The sintered membranes had the form of a truncated cone. This could be a result of non-uniform shrinkage during sintering or, alternately, could come from the formation of a close-packed, cohesive layer of coagulated, rejected nanoparticles in the unfrozen region ahead of the freezing front [1]. The height was always approximately 11 mm. The top and bottom diameters were: 13.5 and 14.0 mm at φ_v_ = 0.064; 13 and 14 mm at φ_v_ = 0.068; and 12.5 and 14.0 mm at φ_v_ = 0.032. A membrane of 5 mm thickness was cut out of each body using a diamond-coated band saw (BS 230 XY-1, Dramet, Kleinmaischeid, Germany), as illustrated in Figure 5. These two parallel cuts run through the cylindrical sintered body and are perpendicular to its top surface. They will not necessarily run exactly parallel to the lamellae and channels running through the sintered body from top to bottom. The reason for this is that the lamellae and pores are not all completely parallel to each other, but rather form domains of parallel pore-groups which are visible in cross-section [1]. In this fashion the apical and basal sides of the resulting membrane (blue colored in Figure 5) run parallel to the direction of ice crystal growth and hence are aligned parallel to the channels running through the membrane. Diffusion through the cut membrane from apical to basal sides, therefore, run perpendicular to the direction of the aligned channels.

### 3.3. Measurement of Steady-State Diffusion

A holding system was prepared for each membrane. The four 5 mm-thick side walls of the membrane (Figure 5A) were first sealed by covering with a thin layer of adhesive. A rubber sealant ring was then glued to both the apical and basal sides of the membrane (Figure 5B) and these covered with two Teflon washers (Figure 5C). The membrane was pre-wetted by drawing water through it at 1 bar pressure difference across the membrane. It was then placed between the ground glass surfaces of the two chambers of a side-by-side glass diffusion cell (Crown Glass, Permegear, Hellertown, PA, USA) thermostatted to 25 ± 0.2 °C. Immediately, the acceptor chamber was filled with 5 mL of water and the donor chamber with 5 mL of an aqueous solution of methylene blue (0.1 mg/5 mL, 0.2 mg/5 mL, or 0.3 mg/5 mL). Both chambers were stirred continuously with stir-bars at 600 rpm for the duration of the experiment. A possible effect of stirring speed on any capillary flow through the membrane cannot be ruled out, but was not examined in this study. At defined time intervals a sample of 0.5 mL of the acceptor solution was removed and its volume replaced with fresh water. The concentration of methylene blue in the acceptor solution, c_a_(*t*), was determined by UV absorption at λ = 664 nm (UV/VIS Genesys 10S; Thermo Fisher scientific, Madison, WI, USA). The result was expressed as a kinetic diffusion profile of m_a_(*t*)/m_d_(0) versus t, where m_a_(*t*) is the amount of methylene blue in the acceptor at time *t* and m_d_(0) is the initial amount of dye in the donor solution. A correction was made to m_a_(*t*) for the loss of dye removed during sampling. At the end of the experiment (*t* = 104 h) a sample of the donor was analyzed for residual dye, m_d_(104 h), and the cell disassembled and the amount of dye in the membrane, m_m_(104 h), extracted in water using three washings each lasting one week. The dye recovery could then be calculated from: m_d_(104 h) + m_m_(104 h) + m_a_(104 h).

### 3.4. Scanning Electron Microscopy (SEM)

After sintering, and before cutting, the intact membranes were examined on an Amray 1810 T SEM (Amray Inc., Bedford, MA, USA) at 20 kV. Each membrane was fixed on an Al stub and Au-sputtered before examination. 

### 3.5. Micro-Computed Tomography

This was performed on a Skyscan 1171 MCT (Skyscan, Kontich, Belgium) which was fitted to an 11 megapixel detector. The X-ray tube ran at 80 kV and 100 µA with an Al 0.5 mm filter removing low-energy X-rays. The sample scanning conditions were: rotation step = 0.25° over 360°; exposure time = 1765 ms per slice; random movement = 10; a resolution resulted of 4.00–4.47 µm per pixel. Each sinogram was reconstructed with the tomographic software N Recon Client and Server 1.6.9 with GPU support (Skyscan, Kontich, Belgium). The channel/pore network was built based on the reconstructed two-dimensional tomography slices using a skeletonization algorithm with the software Amira 6.2 (FEI, Berlin, Germany) [23].

## 4. Conclusions

We draw the following conclusions:

1. Sintered alumina membranes prepared by vacuum-induced surface directional freezing show effective tortuosities, τ* = (*α*/*τ*)^−1^, of substantially greater than unity.
2. The values of τ* increase at higher volume fraction of dispersed phase, at least up to φ_v_ = 0.128. Thus, τ* is inversely dependent on membrane porosity.
3. The highest value of τ* averaged over three donor concentrations examined in this work achieved is 38.4 ± 31.6 (*n* = 10) showing that high effective tortuosities can be achieved in such aligned porous membranes. A potential application as a scaffolding for a surrogate membrane for human stratum corneum is, therefore, revealed.

## Figures and Tables

**Table 1:** Results for calculation of effective tortuosity, τ* = (*α*/*τ*)^−1^ [dimensionless], of the sintered alumina membranes in dependence of slurry volume fraction, φ_v_. The values at each φ_v_ are given for each of three initial donor amounts of dye, m_d_(0), with *n* = 3 or 4. The diffusivity of methylene blue in water at room temperature with D_meth_ = 0.023 cm^2^/h was taken from the literature. The membranes had thickness, *L* = 0.5 cm and surface area, *A* = 0.484 cm^2^.

φ_v_	τ* = (*α*/*τ*)^−1^			
	m_d_(0) = 0.1 mg	m_d_(0) = 0.2 mg	m_d_(0) = 0.3 mg	Mean ± *SD*
0.032	9.73 ± 1.6 (*n* = 4)	9.45 ± 4.1 (*n* = 4)	3.28 ± 0.9 (*n* = 4)	7.48 ± 3.9 (*n* = 12)
0.064	23.1 ± 9.4 (*n* = 4)	21.5 ± 14 (*n* = 4)	24.7± 26 (*n* = 4)	23.1 ± 17.8 (*n* = 12)
0.128	32.8 ± 5.9 (*n* = 3)	69.9 ± 40 (*n* = 3)	18.9 ± 9.3 (*n* = 4)	38.4 ± 31.6 (*n* = 10)
